# Ethyl 2-methyl-4-phenyl­pyrido[1,2-*a*]benzimidazole-3-carboxyl­ate

**DOI:** 10.1107/S1600536811039973

**Published:** 2011-10-05

**Authors:** Yan Qing Ge, Hai Yan Ge, Xiao Qun Cao

**Affiliations:** a School of Chemistry and Chemical Engineering, Taishan Medical College, Tai An, 271016, People’s Republic of China

## Abstract

The title compound, C_21_H_18_N_2_O_2_, was synthesized using a novel tandem annulation reaction between (1*H*-benzimidazol-2-yl)(phen­yl)methanone and (*E*)-ethyl 4-bromo­but-2-enoate under mild conditions. The dihedral angles between the mean planes of the five-membered imidazole ring and the pyridine, benzene and phenyl rings are 0.45 (6), 1.69 (1) and 70.96 (8)°, respectively. In the crystal, mol­ecules are linked through inter­molecular C—H⋯N hydrogen bonds.

## Related literature

For applications of nitro­gen-containing heterocyclic compounds in the agrochemical and pharmaceutical fields, see: Ge *et al.* (2009 ▶). For the synthesis of the title compound, see: Ge *et al.* (2011 ▶). For the structure of 2,7,8-trimethyl-3-eth­oxy­carbonyl-4- phenyl­pyrido[1,2-*a*]benzimidazole, see: Ge *et al.*(2011 ▶). Some pyrido[1,2-*a*]benzimidazole derivatives are of inter­est for their biological activity, such as anti­neoplastic activity and central GABA-A receptor modulators for the treatment of anxiety, see: Badawey & Kappe (1999 ▶). 
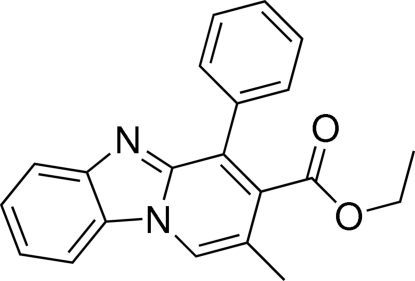


## Experimental

### 

#### Crystal data

C_21_H_18_N_2_O_2_
*M* *_r_* = 330.37
Monoclinic, 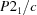

*a* = 10.1176 (13) Å
*b* = 14.9136 (18) Å
*c* = 12.2648 (15) Å
β = 108.487 (2)°
*V* = 1755.1 (4) Å^3^
*Z* = 4
Mo *K*α radiation
μ = 0.08 mm^−1^
*T* = 298 K
0.26 × 0.22 × 0.19 mm

#### Data collection

Bruker SMART APEX CCD diffractometer
Absorption correction: multi-scan (*SADABS*; Sheldrick, 1996 ▶) *T*_min_ = 0.979, *T*_max_ = 0.985
8867 measured reflections
3093 independent reflections
2515 reflections with *I* > 2σ(*I*)
*R*_int_ = 0.021

#### Refinement

*R*[*F* ^2^ > 2σ(*F* ^2^)] = 0.036
*wR*(*F* ^2^) = 0.096
*S* = 1.06
3093 reflections
227 parameters
H-atom parameters constrained
Δρ_max_ = 0.18 e Å^−3^
Δρ_min_ = −0.16 e Å^−3^

### 

Data collection: *SMART* (Bruker, 2005 ▶); cell refinement: *SAINT* (Bruker, 2005 ▶); data reduction: *SAINT*; program(s) used to solve structure: *SHELXS97* (Sheldrick, 2008 ▶); program(s) used to refine structure: *SHELXL97* (Sheldrick, 2008 ▶); molecular graphics: *XP* in *SHELXTL* (Sheldrick, 2008 ▶); software used to prepare material for publication: *SHELXL97*.

## Supplementary Material

Crystal structure: contains datablock(s) I, global. DOI: 10.1107/S1600536811039973/fj2449sup1.cif

Structure factors: contains datablock(s) I. DOI: 10.1107/S1600536811039973/fj2449Isup2.hkl

Supplementary material file. DOI: 10.1107/S1600536811039973/fj2449Isup3.cml

Additional supplementary materials:  crystallographic information; 3D view; checkCIF report

## Figures and Tables

**Table 1:** Hydrogen-bond geometry (Å, °)

*D*—H⋯*A*	*D*—H	H⋯*A*	*D*⋯*A*	*D*—H⋯*A*
C17—H17⋯N2^i^	0.93	2.31	3.2092 (18)	164

Symmetry code: (i) 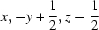.

**Fig. 1.**
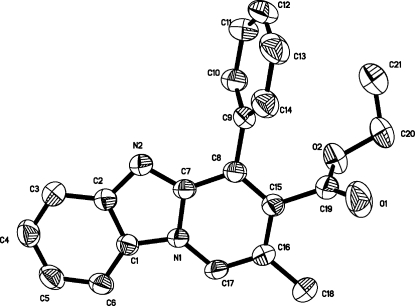
The molecular structure of (I), showing displacement ellipsoids drawn at the 50% probability level.

## supplementary crystallographic information

### Comment

Synthesis of nitrogen-containing heterocyclic compounds has been a subject of great interest due to the wide application in agrochemical and pharmaceutical fields (Ge *et al.*; 2011). Some pyrido[1,2-*a*]benzimidazole derivatives have been of interest for their biological activities, such as antineoplastic activity and central GABA-A receptor modulators for the treatment of anxiety (Badawey *et al.*; 1999). We report here the crystal structure of the title compound, (I) (Fig. 1)

### Experimental

To a 50 ml round-bottomed flask were added (1*H*-benzo[*d*]imidazol-2-yl)(phenyl)methanone (1.00 mmol), (*E*)-ethyl 4-bromobut-2-enoate (2.00 mmol), potassium carbonate (0.28 g, 2.05 mmol) and dry DMF (10 ml). The mixture was stirred at room temperature for 6 h. The solvent was removed under reduced pressure and an product was isolated by column chromatography on silica gel (yield 74%). Crystals of (I) suitable for X-ray diffraction were obtained by allowing a refluxed solution of the product in ethyl acetate to cool slowly to room temperature (without temperature control) and allowing the solvent to evaporate for 3 d

### Refinement

All H atoms were placed in geometrically calculated positions and refined using a riding model with C—H = 0.97 Å (for CH_2_ groups) and 0.96 Å (for CH_3_ groups), their isotropic displacement parameters were set to 1.2 times (1.5 times for CH_3_ groups) the equivalent displacement parameter of their parent atoms.

### Crystal data

**Table:**

C_21_H_18_N_2_O_2_	*F*(000) = 696
*M**_r_* = 330.37	*D*_x_ = 1.250 Mg m^−^^3^
Monoclinic, *P*2_1_/*c*	Mo *K*α radiation, λ = 0.71073 Å
Hall symbol: -P 2ybc	Cell parameters from 4793 reflections
*a* = 10.1176 (13) Å	θ = 2.2–28.2°
*b* = 14.9136 (18) Å	µ = 0.08 mm^−^^1^
*c* = 12.2648 (15) Å	*T* = 298 K
β = 108.487 (2)°	BLOCK, yellow
*V* = 1755.1 (4) Å^3^	0.26 × 0.22 × 0.19 mm
*Z* = 4	

### Data collection

**Table:**

Bruker SMART APEX CCD diffractometer	3093 independent reflections
Radiation source: fine-focus sealed tube	2515 reflections with *I* > 2σ(*I*)
graphite	*R*_int_ = 0.021
φ and ω scans	θ_max_ = 25.0°, θ_min_ = 2.1°
Absorption correction: multi-scan (*SADABS*; Sheldrick, 1996)	*h* = −12→12
*T*_min_ = 0.979, *T*_max_ = 0.985	*k* = −10→17
8867 measured reflections	*l* = −14→14

### Refinement

**Table:**

Refinement on *F*^2^	Secondary atom site location: difference Fourier map
Least-squares matrix: full	Hydrogen site location: inferred from neighbouring sites
*R*[*F*^2^ > 2σ(*F*^2^)] = 0.036	H-atom parameters constrained
*wR*(*F*^2^) = 0.096	*w* = 1/[σ^2^(*F*_o_^2^) + (0.0406*P*)^2^ + 0.4084*P*] where *P* = (*F*_o_^2^ + 2*F*_c_^2^)/3
*S* = 1.06	(Δ/σ)_max_ < 0.001
3093 reflections	Δρ_max_ = 0.18 e Å^−^^3^
227 parameters	Δρ_min_ = −0.16 e Å^−^^3^
0 restraints	Extinction correction: *SHELXL97* (Sheldrick, 2008), Fc^*^=kFc[1+0.001xFc^2^λ^3^/sin(2θ)]^-1/4^
Primary atom site location: structure-invariant direct methods	Extinction coefficient: 0.0077 (13)

### Special details

**Table:**

Geometry. All e.s.d.'s (except the e.s.d. in the dihedral angle between two l.s. planes) are estimated using the full covariance matrix. The cell e.s.d.'s are taken into account individually in the estimation of e.s.d.'s in distances, angles and torsion angles; correlations between e.s.d.'s in cell parameters are only used when they are defined by crystal symmetry. An approximate (isotropic) treatment of cell e.s.d.'s is used for estimating e.s.d.'s involving l.s. planes.
Refinement. Refinement of *F*^2^ against ALL reflections. The weighted *R*-factor *wR* and goodness of fit *S* are based on *F*^2^, conventional *R*-factors *R* are based on *F*, with *F* set to zero for negative *F*^2^. The threshold expression of *F*^2^ > σ(*F*^2^) is used only for calculating *R*-factors(gt) *etc*. and is not relevant to the choice of reflections for refinement. *R*-factors based on *F*^2^ are statistically about twice as large as those based on *F*, and *R*- factors based on ALL data will be even larger.

### Fractional atomic coordinates and isotropic or equivalent isotropic displacement parameters (Å^2^)

**Table:**

	*x*	*y*	*z*	*U*_iso_*/*U*_eq_	
O1	0.10877 (12)	0.07612 (10)	0.79576 (12)	0.0806 (4)	
O2	0.26487 (10)	−0.01607 (7)	0.91064 (10)	0.0565 (3)	
N1	0.57376 (11)	0.22116 (7)	0.87282 (9)	0.0359 (3)	
N2	0.60190 (12)	0.26824 (8)	1.05384 (9)	0.0436 (3)	
C1	0.68800 (13)	0.27812 (9)	0.90225 (11)	0.0375 (3)	
C2	0.70311 (14)	0.30576 (9)	1.01441 (11)	0.0398 (3)	
C3	0.81352 (16)	0.36238 (11)	1.07098 (13)	0.0508 (4)	
H3	0.8262	0.3817	1.1457	0.061*	
C4	0.90241 (16)	0.38855 (11)	1.01303 (14)	0.0545 (4)	
H4	0.9767	0.4260	1.0496	0.065*	
C5	0.88497 (16)	0.36077 (11)	0.90058 (14)	0.0540 (4)	
H5	0.9473	0.3804	0.8640	0.065*	
C6	0.77758 (15)	0.30500 (10)	0.84297 (13)	0.0468 (4)	
H6	0.7654	0.2862	0.7681	0.056*	
C7	0.52624 (13)	0.21772 (9)	0.96751 (11)	0.0365 (3)	
C8	0.40891 (14)	0.16270 (9)	0.95981 (11)	0.0385 (3)	
C9	0.35444 (14)	0.15819 (10)	1.05942 (12)	0.0406 (3)	
C10	0.42867 (17)	0.11386 (11)	1.15914 (13)	0.0511 (4)	
H10	0.5136	0.0872	1.1647	0.061*	
C11	0.3766 (2)	0.10917 (13)	1.25066 (14)	0.0637 (5)	
H11	0.4261	0.0784	1.3170	0.076*	
C12	0.2527 (2)	0.14964 (13)	1.24415 (16)	0.0677 (5)	
H12	0.2186	0.1466	1.3061	0.081*	
C13	0.17917 (19)	0.19447 (12)	1.14640 (17)	0.0634 (5)	
H13	0.0956	0.2225	1.1422	0.076*	
C14	0.22908 (16)	0.19811 (11)	1.05370 (14)	0.0517 (4)	
H14	0.1778	0.2277	0.9869	0.062*	
C15	0.34899 (14)	0.11679 (9)	0.85925 (11)	0.0389 (3)	
C16	0.39968 (14)	0.12299 (9)	0.76289 (11)	0.0400 (3)	
C17	0.51144 (14)	0.17530 (9)	0.77255 (11)	0.0398 (3)	
H17	0.5464	0.1803	0.7112	0.048*	
C18	0.33086 (17)	0.07371 (12)	0.65248 (12)	0.0548 (4)	
H18A	0.3839	0.0823	0.6008	0.082*	
H18B	0.2382	0.0965	0.6179	0.082*	
H18C	0.3265	0.0109	0.6682	0.082*	
C19	0.22564 (15)	0.05809 (10)	0.85009 (12)	0.0443 (3)	
C20	0.15708 (18)	−0.07492 (11)	0.92566 (16)	0.0598 (4)	
H20A	0.1769	−0.1366	0.9109	0.072*	
H20B	0.0673	−0.0587	0.8717	0.072*	
C21	0.15356 (18)	−0.06592 (12)	1.04557 (16)	0.0656 (5)	
H21A	0.2437	−0.0800	1.0985	0.098*	
H21B	0.0855	−0.1064	1.0572	0.098*	
H21C	0.1292	−0.0055	1.0583	0.098*	

### Atomic displacement parameters (Å^2^)

**Table:**

	*U*^11^	*U*^22^	*U*^33^	*U*^12^	*U*^13^	*U*^23^
O1	0.0426 (7)	0.0950 (10)	0.0882 (9)	−0.0126 (6)	−0.0019 (6)	0.0330 (8)
O2	0.0439 (6)	0.0466 (6)	0.0797 (8)	−0.0035 (5)	0.0206 (5)	0.0096 (6)
N1	0.0368 (6)	0.0431 (6)	0.0287 (5)	−0.0039 (5)	0.0119 (5)	0.0010 (5)
N2	0.0455 (7)	0.0532 (7)	0.0345 (6)	−0.0091 (6)	0.0160 (5)	−0.0049 (5)
C1	0.0355 (7)	0.0412 (7)	0.0352 (7)	−0.0016 (6)	0.0105 (6)	0.0041 (6)
C2	0.0404 (7)	0.0438 (8)	0.0347 (7)	−0.0027 (6)	0.0111 (6)	0.0021 (6)
C3	0.0526 (9)	0.0554 (9)	0.0407 (8)	−0.0105 (7)	0.0093 (7)	−0.0024 (7)
C4	0.0449 (8)	0.0561 (10)	0.0555 (10)	−0.0134 (7)	0.0062 (7)	0.0057 (7)
C5	0.0443 (8)	0.0637 (10)	0.0559 (10)	−0.0092 (7)	0.0187 (7)	0.0121 (8)
C6	0.0468 (8)	0.0558 (9)	0.0408 (8)	−0.0046 (7)	0.0182 (7)	0.0061 (7)
C7	0.0375 (7)	0.0438 (8)	0.0295 (6)	−0.0014 (6)	0.0125 (5)	0.0014 (6)
C8	0.0372 (7)	0.0455 (8)	0.0336 (7)	−0.0006 (6)	0.0126 (6)	0.0031 (6)
C9	0.0414 (8)	0.0452 (8)	0.0386 (7)	−0.0096 (6)	0.0176 (6)	−0.0036 (6)
C10	0.0502 (9)	0.0640 (10)	0.0421 (8)	−0.0040 (8)	0.0187 (7)	0.0028 (7)
C11	0.0735 (12)	0.0798 (12)	0.0428 (9)	−0.0143 (10)	0.0257 (8)	0.0046 (8)
C12	0.0809 (13)	0.0801 (13)	0.0603 (11)	−0.0246 (11)	0.0483 (10)	−0.0144 (10)
C13	0.0583 (10)	0.0676 (11)	0.0788 (13)	−0.0100 (9)	0.0425 (10)	−0.0138 (10)
C14	0.0472 (8)	0.0547 (9)	0.0572 (9)	−0.0036 (7)	0.0224 (7)	−0.0012 (7)
C15	0.0365 (7)	0.0430 (8)	0.0362 (7)	−0.0008 (6)	0.0100 (6)	0.0037 (6)
C16	0.0421 (8)	0.0440 (8)	0.0320 (7)	−0.0008 (6)	0.0090 (6)	0.0008 (6)
C17	0.0445 (8)	0.0480 (8)	0.0278 (7)	−0.0012 (6)	0.0129 (6)	0.0000 (6)
C18	0.0591 (10)	0.0625 (10)	0.0397 (8)	−0.0102 (8)	0.0113 (7)	−0.0080 (7)
C19	0.0394 (8)	0.0531 (9)	0.0398 (7)	−0.0045 (7)	0.0115 (6)	−0.0019 (7)
C20	0.0554 (10)	0.0456 (9)	0.0834 (12)	−0.0115 (7)	0.0291 (9)	0.0012 (8)
C21	0.0583 (10)	0.0596 (11)	0.0838 (13)	0.0024 (8)	0.0294 (10)	0.0088 (9)

### Geometric parameters (Å, °)

**Table:**

O1—C19	1.1894 (18)	C10—C11	1.385 (2)
O2—C19	1.3211 (18)	C10—H10	0.9300
O2—C20	1.4564 (18)	C11—C12	1.371 (3)
N1—C17	1.3733 (16)	C11—H11	0.9300
N1—C1	1.3868 (17)	C12—C13	1.368 (3)
N1—C7	1.3917 (16)	C12—H12	0.9300
N2—C7	1.3270 (17)	C13—C14	1.384 (2)
N2—C2	1.3818 (17)	C13—H13	0.9300
C1—C6	1.3887 (19)	C14—H14	0.9300
C1—C2	1.3973 (19)	C15—C16	1.4328 (19)
C2—C3	1.397 (2)	C15—C19	1.4992 (19)
C3—C4	1.368 (2)	C16—C17	1.3477 (19)
C3—H3	0.9300	C16—C18	1.5036 (19)
C4—C5	1.397 (2)	C17—H17	0.9300
C4—H4	0.9300	C18—H18A	0.9600
C5—C6	1.373 (2)	C18—H18B	0.9600
C5—H5	0.9300	C18—H18C	0.9600
C6—H6	0.9300	C20—C21	1.488 (3)
C7—C8	1.4214 (19)	C20—H20A	0.9700
C8—C15	1.3722 (19)	C20—H20B	0.9700
C8—C9	1.4926 (19)	C21—H21A	0.9600
C9—C14	1.383 (2)	C21—H21B	0.9600
C9—C10	1.384 (2)	C21—H21C	0.9600
			
C19—O2—C20	118.17 (12)	C13—C12—H12	120.0
C17—N1—C1	130.26 (11)	C11—C12—H12	120.0
C17—N1—C7	123.15 (11)	C12—C13—C14	120.04 (16)
C1—N1—C7	106.58 (10)	C12—C13—H13	120.0
C7—N2—C2	104.76 (11)	C14—C13—H13	120.0
N1—C1—C6	131.99 (13)	C9—C14—C13	120.62 (16)
N1—C1—C2	105.00 (11)	C9—C14—H14	119.7
C6—C1—C2	122.99 (13)	C13—C14—H14	119.7
N2—C2—C3	129.47 (13)	C8—C15—C16	122.51 (12)
N2—C2—C1	111.30 (12)	C8—C15—C19	118.60 (12)
C3—C2—C1	119.21 (13)	C16—C15—C19	118.88 (12)
C4—C3—C2	117.93 (14)	C17—C16—C15	118.34 (12)
C4—C3—H3	121.0	C17—C16—C18	119.87 (13)
C2—C3—H3	121.0	C15—C16—C18	121.79 (13)
C3—C4—C5	122.03 (15)	C16—C17—N1	120.08 (12)
C3—C4—H4	119.0	C16—C17—H17	120.0
C5—C4—H4	119.0	N1—C17—H17	120.0
C6—C5—C4	121.30 (14)	C16—C18—H18A	109.5
C6—C5—H5	119.4	C16—C18—H18B	109.5
C4—C5—H5	119.4	H18A—C18—H18B	109.5
C5—C6—C1	116.54 (14)	C16—C18—H18C	109.5
C5—C6—H6	121.7	H18A—C18—H18C	109.5
C1—C6—H6	121.7	H18B—C18—H18C	109.5
N2—C7—N1	112.36 (11)	O1—C19—O2	124.91 (14)
N2—C7—C8	129.73 (12)	O1—C19—C15	124.47 (14)
N1—C7—C8	117.91 (11)	O2—C19—C15	110.62 (12)
C15—C8—C7	117.99 (12)	O2—C20—C21	108.91 (14)
C15—C8—C9	122.73 (12)	O2—C20—H20A	109.9
C7—C8—C9	119.27 (12)	C21—C20—H20A	109.9
C14—C9—C10	118.86 (14)	O2—C20—H20B	109.9
C14—C9—C8	120.66 (13)	C21—C20—H20B	109.9
C10—C9—C8	120.48 (13)	H20A—C20—H20B	108.3
C9—C10—C11	120.09 (16)	C20—C21—H21A	109.5
C9—C10—H10	120.0	C20—C21—H21B	109.5
C11—C10—H10	120.0	H21A—C21—H21B	109.5
C12—C11—C10	120.43 (17)	C20—C21—H21C	109.5
C12—C11—H11	119.8	H21A—C21—H21C	109.5
C10—C11—H11	119.8	H21B—C21—H21C	109.5
C13—C12—C11	119.93 (15)		
			
C17—N1—C1—C6	−2.2 (2)	C15—C8—C9—C10	109.59 (17)
C7—N1—C1—C6	178.59 (15)	C7—C8—C9—C10	−71.49 (18)
C17—N1—C1—C2	179.46 (13)	C14—C9—C10—C11	0.7 (2)
C7—N1—C1—C2	0.20 (14)	C8—C9—C10—C11	−179.29 (14)
C7—N2—C2—C3	−177.76 (15)	C9—C10—C11—C12	−1.2 (3)
C7—N2—C2—C1	0.44 (16)	C10—C11—C12—C13	0.5 (3)
N1—C1—C2—N2	−0.40 (15)	C11—C12—C13—C14	0.7 (3)
C6—C1—C2—N2	−178.98 (13)	C10—C9—C14—C13	0.5 (2)
N1—C1—C2—C3	178.00 (13)	C8—C9—C14—C13	−179.52 (14)
C6—C1—C2—C3	−0.6 (2)	C12—C13—C14—C9	−1.2 (3)
N2—C2—C3—C4	178.25 (15)	C7—C8—C15—C16	−0.4 (2)
C1—C2—C3—C4	0.2 (2)	C9—C8—C15—C16	178.50 (13)
C2—C3—C4—C5	0.3 (2)	C7—C8—C15—C19	179.67 (12)
C3—C4—C5—C6	−0.5 (3)	C9—C8—C15—C19	−1.4 (2)
C4—C5—C6—C1	0.1 (2)	C8—C15—C16—C17	0.7 (2)
N1—C1—C6—C5	−177.72 (14)	C19—C15—C16—C17	−179.38 (13)
C2—C1—C6—C5	0.4 (2)	C8—C15—C16—C18	−178.70 (14)
C2—N2—C7—N1	−0.31 (15)	C19—C15—C16—C18	1.2 (2)
C2—N2—C7—C8	179.29 (14)	C15—C16—C17—N1	−0.1 (2)
C17—N1—C7—N2	−179.25 (12)	C18—C16—C17—N1	179.34 (13)
C1—N1—C7—N2	0.07 (15)	C1—N1—C17—C16	−179.97 (13)
C17—N1—C7—C8	1.10 (19)	C7—N1—C17—C16	−0.8 (2)
C1—N1—C7—C8	−179.58 (12)	C20—O2—C19—O1	−8.9 (2)
N2—C7—C8—C15	179.97 (14)	C20—O2—C19—C15	171.09 (13)
N1—C7—C8—C15	−0.45 (19)	C8—C15—C19—O1	105.81 (18)
N2—C7—C8—C9	1.0 (2)	C16—C15—C19—O1	−74.1 (2)
N1—C7—C8—C9	−179.42 (12)	C8—C15—C19—O2	−74.15 (16)
C15—C8—C9—C14	−70.36 (19)	C16—C15—C19—O2	105.95 (15)
C7—C8—C9—C14	108.56 (16)	C19—O2—C20—C21	−106.00 (16)

### Hydrogen-bond geometry (Å, °)

**Table:**

*D*—H···*A*	*D*—H	H···*A*	*D*···*A*	*D*—H···*A*
C17—H17···N2^i^	0.93	2.31	3.2092 (18)	164

Symmetry codes: (i) *x*, −*y*+1/2, *z*−1/2.
